# A Statewide Observational Assessment of the Pedestrian and Bicycling Environment in Hawaii, 2010

**Published:** 2011-12-15

**Authors:** Jay E. Maddock, Vickie Ramirez, Miaoxuan Zhang, I. Made Brunner, Katie M. Heinrich

**Affiliations:** University of Hawaii at Manoa, Honolulu, Hawaii; University of Hawaii at Manoa, Honolulu, Hawaii; University of Hawaii at Manoa, Honolulu, Hawaii; Kansas State University, Manhattan, Kansas

## Abstract

**Introduction:**

Walking and bicycling are important but underused modes of transportation in the United States. Road design influences how much walking and bicycling takes place along streets and roads. Currently, numerous national policy initiatives, including Safe Routes to School and Complete Streets, are attempting to improve pedestrian and bicycling infrastructure and "friendliness." However, no state has completed a systematic assessment of its streets to determine how amenable they are to walking and bicycling. Our statewide study was undertaken to assess how accessible and friendly Hawaii roads are to these 2 activities.

**Methods:**

We randomly selected street segments in Hawaii's 4 counties and then completed objective assessments using the Pedestrian Environmental Data Scan. We audited 321 segments, and interrater reliability was adequate across all measures. Streets were coded as high (42.4%) or low capacity (57.6%) depending on how much vehicular traffic the street was designed to accommodate. Outcome measures included street accommodations (ie, sidewalks and crossing aids) and pedestrian and bicyclist use.

**Results:**

Most high-capacity streets had sidewalks (66%). These sidewalks were usually in good condition, contiguous, and had traffic control devices and pedestrian signals. Most low-capacity roads did not have sidewalks (63.4%). Bicycling facilities were limited (<10%) on both types of roads. Pedestrian and bicycle traffic was related to mixed use, including both residential and retail space, and to pedestrian and bicycling infrastructure.

**Conclusions:**

Road segments in Hawaii with more infrastructure and types of use, including single-family houses, apartment complexes, restaurants, office buildings, and industrial buildings, are used more by pedestrians and bicyclists.

## Introduction

Walking and bicycling are underused modes of transportation in the United States (1). These forms of active transportation increase physical activity, prevent or mitigate obesity, reduce traffic and pollution, and reduce carbon dioxide emissions (2-4). Despite these numerous benefits, walking and bicycling accounted for less than 5% of commuting trips in the United States in 1990 (1). During the past 2 decades, federal, state, and local governments have renewed their efforts to increase these 2 forms of active transportation. Major federal funding and policy initiatives, such as the Transportation Equity Act for the 21st Century and the Safe, Accountable, Flexible, Efficient Transportation Equity Act: A Legacy for Users, provided funding to improve the nation's pedestrian and bicycling infrastructure (1). Awareness of the rising problem of obesity has further spurred action at all levels of government. For instance, by 2010, there were 48 US municipalities with Complete Streets (www.completestreets.org/) ordinances and another 99 with Complete Streets resolutions (5). Walking and bicycling trips increased by 25% from 1999 to 2009, yet still accounted for fewer than 12% of total trips taken (1). At state and city levels, increases in active transportation were linked to less self-reported obesity and diabetes (6).

The environment influences use of active transportation. A recent systematic review of the effect of the street and road environment on cycling found that dedicated cycling routes, separation from traffic, high population density, short trip distance, and Safe Routes to School (http://safety.fhwa.dot.gov/saferoutes/) projects were positively related to the amount of cycling (7). Similar results were found for walking where population density, distance to nonresidential destinations, land-use mix (ie, single-family houses, apartment complexes, restaurants, office buildings, and industrial buildings), connectivity, and access to open space were all positive predictors (8). Most studies focused on urban or suburban areas; data are limited for rural areas (9).

During the past 2 decades, the rise of obesity has become a major public health concern (10). Creating safe communities that encourage physical activity is one of the main recommendations from the Centers for Disease Control and Prevention (CDC) for obesity prevention (11). The goal of federal funding initiatives, including Safe Routes to School and the Complete Streets movement, as well as numerous state and local initiatives, has been to improve environments to make them more conducive to walking and bicycling. However, little has been done to systematically measure the effect of these efforts on changes at the street-segment level. The objective of our study was to provide a statewide assessment of the pedestrian and bicycling environment in Hawaii.

## Methods

Our study consisted of 2 phases. First, we randomly selected a statewide sample of street segments. Second, we directly assessed each segment for its pedestrian and bicycling environment using a reliable audit tool.

### Segment selection

We randomly selected road segments from the US Geological Survey's major roads maps (provided by the state of Hawaii Geographic Information Systems [GIS] [Environmental Systems Research Institute, Inc, Redlands, California] program) using ArcGIS (Environmental Systems Research Institute, Inc, Redlands, California) to get a representative sample across the state of Hawaii. Hawaii has 4 counties. Honolulu, the most populous, accounts for more than 80% of the state's population (12). The other 3 counties, Maui, Kauai, and Hawaii, are more rural but are rapidly becoming urban (12). We selected roads from the islands of Maui, Oahu (Honolulu County), Kauai, and Hawaii that together accounted for 94.7% of all major roads statewide. We defined roads as major if they were 40 to 700 feet long, ran through a census tract with more than 1 person per square mile, were not an interstate highway, and did not intersect federal lands (eg, national parks, military installations). We divided all roads into segments of 700 feet or less to create units that were feasible for measurement. This process generated 301 eligible segments on Kauai, 1,274 on Oahu, 360 on Maui, and 686 on Hawaii. Our final segment selection was done randomly based on the population on each island, yielding 10 on Kauai, 50 on Oahu, 20 on Maui, and 30 on the island of Hawaii. We manually screened all selected road segments using ArcGIS to ensure that none of the selected segments was located in an inaccessible or unsafe place, such as a state highway without pedestrian access. We then selected 2 adjoining segments for each major road segment using a defined directional protocol (Figure). This yielded a target sample size of 330 road segments.

**Figure.**
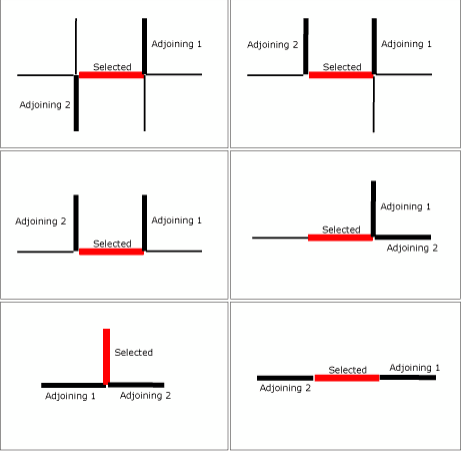
Method for selecting adjoining street samples, a statewide observational assessment of the pedestrian and bicycling environment in Hawaii.Legend: GIS was used to randomly select the red street segments. Two adjoining street segments (bold black lines) were then randomly selected according to availability.

### Segment audits

We assessed each selected segment using the Pedestrian Environment Data Scan (PEDS) (13). PEDS is a comprehensive environmental audit methodology that assesses numerous factors, including land use, sidewalk presence, traffic volume, parking, speed limits, lighting, tree coverage, and several other factors. Seventy-nine objective and 4 subjective factors are measured for each segment to assess the walking and bicycling environments (13). The PEDS instrument was subject to rigorous testing and development and was found to have adequate intra- and interrater reliability across measurements (13). All auditors were rigorously trained before data collection. Training consisted of classroom-based instruction, including reviewing PowerPoint slides of the different codes and conditions followed by a question and answer period, and physical assessments of street segments surrounding the University of Hawaii (14). We continued the training until observers achieved a high level of interrater reliability.

Our trained observers conducted audits between April and July of 2010 between 8 AM and 6 PM. They worked in pairs to ensure safety and to assess interrater reliability. After each segment, observers switched audit sheets and reviewed their counterparts' answers to ensure that all answers were completed and readable. In 3 cases, observers were unable to find the selected road or felt that the road was too dangerous (eg, heavy traffic with no shoulder area) to complete the audit. We removed these 3 streets and their adjoining segments (n = 9) from the target sample. We coded roads as high capacity if they had more than 1 lane in each direction. All other roads were coded as low capacity.

Our research team had previously assessed all 4 counties for policies supporting active community environments, including mixed use, sidewalk ordinances, and bike lanes (15). We found Honolulu (n = 15) and Kauai (n = 14) to have more policies than Hawaii (n = 4) and Maui (n = 3) (14). In this study, we compared all assessment data by county. This analysis also allowed us to examine urban and rural differences. Honolulu County is designated urban by the US Census Bureau, and all other counties are designated rural.

We assessed road use for vehicle traffic, pedestrians, and cyclists at the same time that we did the PEDS assessments. We had observers visually draw a line across the middle of the street segment from 1 side of the street to the other. For 3 minutes, they counted the number of vehicles that crossed the line. In the next 3 minutes they counted all pedestrians and cyclists that crossed the line. Because of limited resources, we could conduct these counts at only 1 time during the day. To help control for this, we stratified our observations throughout the day by island and by road capacity. In addition, we created a mixed-use index by summing the different types of use for each segment. Single-family houses, apartment complexes, restaurants, office buildings, and industrial buildings were each given 1 point if they were present in the segment. These were summed to create an index of  0-5.

### Data analysis

We entered all data in PASW Statistics 18.0 (IBM Corp, Somers, New York) for analysis. We calculated Cohen's k to assess interrater reliability. We reported the data using mean, standard deviation, and percentage. We reported differences by categories using χ^2^ , *t*-test, or analysis of variance test.

## Results

### Interrater reliability

Overall, 321 (97.3%) street segments were assessed in the study. Interrater reliability was excellent across almost all assessment items (median κ = 0.96). Only 2 items had k scores lower than 0.40. These were subjective items measuring cycling attractiveness (κ = 0.16) and walking safety (κ = 0.39). These items are not reported in this study because of their low level of interrater reliability. All percentages indicate the proportion of the 321 segments that contained that item.

### Pedestrian and bicycle accommodations by road capacity

Fewer than half of the roads we studied were coded as high capacity (42.6%). High-capacity roads were more common in Honolulu County (59.6%) than in Maui (40.4%), Hawaii (28.9%), or Kauai (20.0%) counties.  Motor vehicle traffic (ie, number of vehicles per segment counted in 3 minutes) was greater on high-capacity roads (mean, 84.5; SD, 50.5) than on low-capacity roads (mean, 13.0; SD, 15.6) (*P* < .001). We observed more pedestrians and cyclists per segment on high-capacity roads than on low-capacity roads (mean, 6.3 vs mean, 1.2, pedestrians; (mean, 1.0 vs mean, 0.2, cyclists). High-capacity road segments contained more apartment complexes, restaurants, industrial buildings, and recreational space than low-capacity roads did and had fewer single-family homes and vacant lots (Table 1). Pedestrian accommodations, including crosswalks, pedestrian signals, and sidewalks, were observed more often on high-capacity roads than on low-capacity roads (*P* < .05). We seldom observed speed bumps (3.4%), pedestrian paddles (0.5%), curb extensions (0.3%), or flashing warning lights (0.6%). High-capacity roads had significantly more bicycle route signs and bicycle parking; however, bicycle accommodations of any type were rare across the state: 97.8% of low-capacity roads and 75.0% of high-capacity roads had none at all (*P* < .001) (Table 1).

### Pedestrian and bicycle accommodations by county

Honolulu County had significantly more sidewalks (71.6%) than Maui (45.6%), Hawaii (17.8%), or Kauai (26.7%) (Table 2). Honolulu also had more crossing aids (78.5%) per segment than Maui (50.9%), Hawaii (25.6%), or Kauai (23.3%). However, both Maui (19.3%) and Honolulu (16.0%) had more bicycle accommodations than Kauai (3.3%) or Hawaii (3.3%).

### Pedestrian and cyclist use by accommodations

We observed pedestrians significantly more often on road segments where crossing aids were present (mean, 5.8; SD, 11.0) than absent (mean, 0.5; SD, 0.5). This difference in pedestrian traffic was also observed when the roads were sorted by capacity. High-capacity with accommodations had more pedestrian traffic than those without accommodations (mean, 7.7; SD, 13.2 vs mean, 0.8; SD, 2.6). Pedestrian traffic was also higher on low-capacity roads with aids compared with those without (mean, 2.5; SD, 4.6 vs mean, 0.5; SD, 1.0 ). Bicyclists were also observed more often on roads with bicycle accommodations (mean, 1.3; SD, 1.87) than on those without (mean, 0.4; SD, 1.00). This was also true for high-capacity roads with such accommodations compared with those without (mean, 1.5; SD, 1.94 vs mean, 0.8; SD, 1.40) (Table 3). We did not examine low-capacity roads for bicycle traffic in relation to bicycle accommodations because only 4 low-capacity roads had any. All *P*-values are less than .05.

Pedestrians were more often observed in Honolulu (mean, 5.5; SD 11.2) and Maui (mean, 2.8; SD, 7.9) than in Hawaii (mean, 1.3; SD, 2.7) or Kauai (mean, 0.8; SD, 1.9) (*P* < .05. Bicyclists were observed significantly more often in Honolulu (mean, 0.72; SD, 1.43) than in Kauai (mean, 0.1; SD, 0.53) (*P* < .05). Pedestrians were more likely to be observed in the afternoon (mean, 5.3; SD, 23.7) than in the morning (mean, 2.5; SD, 5.0) (*P* < .001). No differences were observed for bicyclists by time of day.

Finally, we examined pedestrian and bicycle traffic by level of mixed use. Pedestrians were observed significantly more often in areas where there were 2 or more types of mixed use (mean 0.13, SD 0.52, for 0 types of mixed use vs mean 6.17, SD, 9.39 for 2 types of mixed use and mean 8.71, SD 9.39 for 4 types of mixed use). The number of bicyclists also significantly increased by the level of mixed use of the segment (mean 0.20, SD, 0.41, for 0 types of mixed use vs 0.70, SD 1.34, for 2 types of mixed use and mean 1.43, SD 1.27, for 4 types of mixed use).

## Discussion

Consistent with previous work, our results showed a high level of interrater reliability. High-capacity roads across the state had a good level of pedestrian support; the majority had sidewalks, crosswalks, speed limit signs, and pedestrian signals. Low-capacity roads were much less likely to have pedestrian accommodations. Overall, curb extensions, flashing warning lights, and pedestrian paddles were underused tools in Hawaii: less than 1% of observed segments had them. Cycling accommodations were rare across both road types; almost 90% of all 321 segments studied had no accommodations for cyclists.

Pedestrian and cycling infrastructure varied widely among the counties. Not surprisingly, Honolulu County, which had the highest number of written policies, had the most supportive infrastructure and the most use. However, Kauai, which had the second highest number of written policies, had few pedestrian and cycling accommodations. Maui had few written policies but good infrastructure, especially for bicycling. In 1 study, a large discrepancy was found between the policies reported by the county planning department and actual written policies (16). These data may explain the difference between written policy and practice at the planning department level. More research is needed to understand this discrepancy.

Previous research has shown that mixed-use design and pedestrian and bicyclist infrastructure are related to overall use of bicycling and walking as a means of transportation (3,6,7). Large effects were seen for several variables. For example, the number of pedestrians observed increased 11-fold when crossing aids were present in the road segment. Bicycling increased 3-fold when the segment had any bicycling accommodations. Mixed use also showed a strong effect on pedestrian use and a more modest one for bicycling. This finding is consistent with previous studies that show a 2-fold increase in use of active transportation in neighborhoods with many pedestrian accommodations compared with neighborhoods with few (16). These baseline findings provide support for Complete Streets-style policy changes that aim to accommodate all users through infrastructure improvements.

In our study, high-capacity roads showed consistently higher levels of pedestrian and bicycling infrastructure. However, many of the routes that people take to get to and from destinations include a mix of both high- and low-capacity roads. The decision whether to use active transportation or to drive many be made based on the least desirable segment on a route. This observation may be especially important for young children and for those with impaired mobility (17,18).

This study has some limitations. The number of segments selected was limited by a combination of time, resources, and observer safety. The sample size of more than 300 segments for the state appears to be robust, but in smaller, more rural counties, the estimates may be subject to change depending on the sampling. The methodology also does not allow for the measurement of connectivity between locations. This could be addressed in the future by using the network analysis tool in ArcGIS. Day and time of day of observation influenced counts of pedestrians and bicyclists. All observations occurred on weekdays, but strict adherence to time-of-day sampling was not feasible. More pedestrians were observed in the afternoon than in the morning. The observation of high- and low-capacity roads across counties was stratified to include assessments at different times and days of the week, but any particular road would likely change dramatically during the course of the day or week. The standard deviation was greater than the mean for pedestrians and bicyclists due to the number of areas where no pedestrians or bicyclists were observed in a 3-minute period while some sections had many pedestrians and bicyclists. This study represents only a single time point. Thus, it cannot establish causality or temporal relationships between pedestrian and bicycle use and the built environment. It is possible that pedestrian crossing aids and bicycle accommodations were built in areas where high demand and use already existed. Future administrations of our surveillance system will help address these uncertainties and also provide the potential to assess infrastructure and usage changes over time.

Long-term changes, such as increases in physical activity or in the number of trips pedestrians or bicyclists take, will be preceded, theoretically, by supportive changes in the pedestrian and bicycling infrastructure. Surveillance systems that collect data on a systematic and regular basis are needed to assess the effect of policy changes on the pedestrian and bicycling infrastructure at the local, state, and national level. In our study we developed and implemented the first statewide assessment of the pedestrian and bicycling environment in Hawaii. The results provide a picture of where Hawaii is now in terms of support in these areas and where changes can be made. Future iterations of this assessment along with continued policy tracking can assess the effectiveness of policy changes at the state and county level. For instance, shortly after the data collection in this study was completed, the Kauai County Council passed a Complete Streets resolution. Our assessment tool could be used to determine if this resolution actually makes a measurable difference in the pedestrian and bicycling infrastructure. Our methodology also provides a framework for other states in developing a surveillance system for pedestrian and bicycling infrastructure. However, several additional features, including the timing of intervals between assessments and the number of street segments needed to assess change, will need to be developed further before this can occur.

## Figures and Tables

**Table 1:** Road Capacity Characteristics and Pedestrian and Bicycle Accommodations, Statewide Observational Assessment of the Pedestrian and Bicycling Environment in Hawaii, 2010

**Road Characteristic**	**All Roads, %** (n = 319)^a^	**High-Capacity Roads^b^, (n = 136)**	**Low-Capacity Roads^c^, (n = 183)**	***P* Value by Road Capacity^d^**
**Types of dwellings or land use present**				
Single-family home	67.9	39.7	89.1	< .001
Apartment complexes	15.6	22.8	10.4	.003
Office buildings	32.1	50.7	17.5	<.001
Restaurants, cafes	26.2	47.1	10.4	<.001
Industrial buildings	5.6	9.6	2.2	.005
Vacant lots	28.0	22.1	32.8	.04
Recreation areas	8.1	13.2	4.4	.006
**Pedestrian accommodations, (*P* <.05)**				
Sidewalks	47.5	68.4	31.7	<.001
Speed limit sign	56.4	52.7	59.1	.26
Crosswalks	54.7	82.4	34.1	< .001
Pedestrian paddles	0.3	0	0.5	.39
Pedestrian signals	32.0	61.0	10.4	<.001
Curb extensions	0.3	0	0.5	.39
Pedestrian crossing warning signs	11.6	15.4	8.7	.07
Flashing warning lights	0.6	0.7	0.5	.83
No pedestrian accommodations	46.7	20.6	66.1	<.001
**Bicycling accommodations**				
Bicycle route signs	5.3	10.3	1.6	<.001
Striped bicycle lanes	5.0	10.3	1.1	<.001
Bicycle parking	2.8	6.6	0	<.001
Bicycle crossing warning signs	0	0	0	NA
"Share the Road" signs	0.9	1.5	0.5	.40
Separate bicycle path	0.9	2.2	0	.04
No bicycle accommodations	88.1	75.0	97.8	<.001

Abbreviation: NA, not applicable.

a Two cases were excluded because of missing data in the road capacity field.

b Roads with more than 1 lane in each direction.

c Roads with only 1 lane in each direction.

d Compares difference in characteristics by road capacity. Calculated by using &chi^2^ test.

**Table 2:** Pedestrian and Bicycle Accommodations, by County, Statewide Observational Assessment of the Pedestrian and Bicycling Environment in Hawaii, 2010

**Road Characteristic**	**Honolulu, % (n = 144 segments)**	**Maui, % (n = 57 segments)**	**Hawaii, % (n = 90 segments)**	**Kauai, % (n = 30 segments)**	***P *Value by County^a^**
**Pedestrian accommodations^b^**					
Sidewalks	71.6	45.6	17.8	26.7	<.001
Speed limit sign	55.5	68.4	47.8	60.0	.10
Crosswalks	79.1	47.4	31.1	20.7	<.001
Pedestrian signals	52.1	24.6	11.1	16.7	<.001
Pedestrian crossing warning signs	13.9	12.3	8.9	6.7	.55
No crossing aids in the segment	21.5	49.1	74.4	76.7	<.001
**Bicycling accommodations**					
Bicycle route signs	6.3	14.0	0	0	<.001
Striped bicycle lanes	6.3	12.3	0	0	<.001
Bicycle parking	5.6	1.8	0	0	.05
No bicycle accommodations	84.0	80.7	96.7	96.7	<.001

a Compares difference in characteristics by county. Calculated by using χ^2^ test.

b Accommodations that occurred in fewer than 1% of road segments overall were not included in this analysis.

**Table 3:** Pedestrian and Bicycle Use by Street Segment (N = 321) in Relation to Pedestrian and Bicycle Accommodations, Statewide Observational Assessment of the Pedestrian and Bicycling Environment in Hawaii, 2010

**Presence of Pedestrian and Bicycle Accommodations**	Present, Mean (SD)	Absent, Mean (SD)	Statistical Test	P Value
**Pedestrian accommodations**				
All roads, pedestrians per segment^a^	5.8 (11.0)	0.5 (1.4)	*t* (313) = 5.75	<.001
High capacity roads, pedestrians per segment	7.7 (13.2)	0.8 (2.6)	*t*(130) = 2.76,	.02
Low- capacity roads, pedestrians per segment	2.5 (4.6)	0.5 (1.0)	*t*(179) = 4.78	<.001
**Bicycle accommodations**				
All roads, cyclists per segment	1.3 (1.9)	0.4 (1.0)	*t*(313) = 4.45	<.001
High-capacity roads^b^, bicyclists per segment	1.5 (1.9)	0.81 (1.4)	*t*(_130_) = 2.03	.02

a Road segments randomly selected from US Geological Survey major road maps by using Geographic Information Systems (Environmental Systems Research Institute, Inc, Redlands, California).

b Only 4 low-capacity roads had any bicycle accommodations, so they are not included in this table.
